# Flower Size as an Honest Signal in Royal Irises (*Iris* Section *Oncocyclus*, Iridaceae)

**DOI:** 10.3390/plants12162978

**Published:** 2023-08-18

**Authors:** Sissi Lozada-Gobilard, Nadine Nielsen, Yuval Sapir

**Affiliations:** 1 The Botanical Garden, School of Plant Sciences and Food Security, G.S. Wise Faculty of Life Science, Tel Aviv University, Tel Aviv 69978, Israel; nadinen@mail.tau.ac.il (N.N.); sapiry@tauex.tau.ac.il (Y.S.); 2 Biodiversity Unit, Department of Biology, Lund University, 223 62 Lund, Sweden

**Keywords:** honest signal, floral traits, flower size, color signal, shelter reward, fitness, Royal irises, *Oncocyclus*, endemic plant species, morphometrics

## Abstract

Flower traits, such as flower size or color changes, can act as honest signals indicating greater rewards such as nectar; however, nothing is known about shelter-rewarding systems. Large flowers of Royal irises offer overnight shelter as a reward to *Eucera* bees. A black patch might signal the entrance to the tunnel (shelter) and, together with the flower size, these might act as honest signals. We hypothesize that larger flowers and black patches indicate larger tunnels, and larger tunnels will increase pollinator visits, enhancing the plants’ reproductive success. We measured seven species in a controlled environment and two species from three natural populations varying in flower size. Fruit and seed sets were assessed in these natural populations. We found a positive correlation between the flower, patch size, and tunnel volume, suggesting that the flowers and patch size act as honest signals, both under controlled conditions and in the wild. However, in natural populations, this positive relationship and its effect on fitness was population-specific. Flower size increased the fitness in YER *I. petrana*, and interactions between flower/patch size and tunnel size increased the fitness in YER and *I. atropurpurea* NET populations. This suggests that the honesty of the signal is positively selected in these two populations. This study supports the hypothesis that pollinator-mediated selection leads to the honest signaling of flower advertisement.

## 1. Introduction

Plant–pollinator interactions are a typical example of a mutualistic relationship. Plants use flower traits for advertisement and the attraction of pollinators [1]. Plants benefit from pollinators transporting pollen from flower to flower to assure their reproduction [2], and in return, pollinators receive a reward, commonly food (e.g., nectar or pollen). When a flower trait is positive correlated to the reward, it is known as honest signaling [3,4]. Honest signaling, which is a positive relationship between the signal and reward (reviewed in [5]), has been found within species [6,7], communities [8], and across ecosystems [9]. They are mostly found between floral advertisement (e.g., flower size, color, etc.) and nectar reward [10,11], but have also been found in pollen [12,13] and resin rewarding systems [14].

Plants changing color once they have been pollinated is the most obvious example of an honest signal [3,15]. Color changes in nectar guides were shown to be reliable signals to pollinators, enhancing the plants’ reproductive success [16]. Larger nectar guides can increase pollen deposition [17] and be positively selected by pollinators [18]. However, the size and shape of nectar guides could cause disruptive selection when extreme phenotypes are selected by distinct pollinator groups (i.e., insects and birds) [19]. In some nectarless species such as *Oncocyclus* irises, dark-colored flowers mimic shelters [20,21], and a darker spot at the entrance of the shelter may serve as guide for pollinators [22], functioning similarly to nectar guides.

The flower size can be a visual signal of higher-energy rewards such as nectar or pollen, and therefore, are another example of an honest signal [10,23,24,25]. Larger flowers are easier to detect from a distance [26], and thus, the flower size might be positively selected by pollinators [27,28,29,30]. As larger flowers attract naïve pollinators, the positive feedback between the visual cue and the reward enhances pollinator learning [26,31,32].

**Figure 1:**
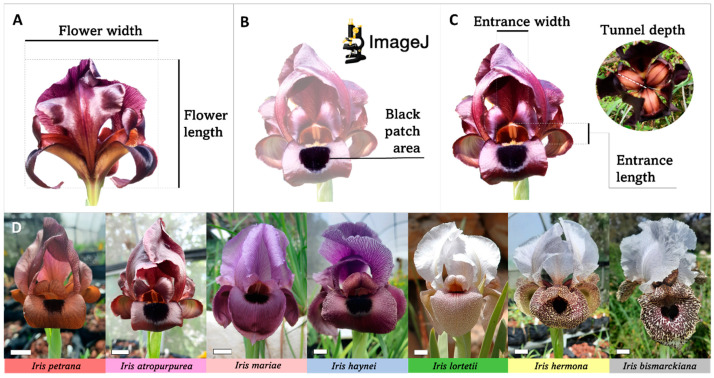
Flower size, black patch size, and tunnel volume measurements. Flower size was calculated by multiplying the width and length (**A**). Black patch size was calculated from digital photographs using ImageJ (**B**). Tunnel volume was calculated by multiplying the width and length of the entrance and the tunnel depth (**C**). Examples of flowers corresponding to each species measured under controlled conditions of the TAUBG (**D**). Scale bar in photographs = 1 cm. Measurements (**A**–**C**) are demonstrated on *Iris atropurpurea*.

Royal irises (*Iris* section *Oncocyclus*) possess exceptionally large, beautiful flowers up to 12 cm in diameter, making them among the largest flowers in the flora of the Middle East [33,34]. These irises are endemic to the Middle East and serve as models for the evolutionary processes of speciation, pollination, and ecology [34,35,36,37,38]. They typically produce one large flower per stem, and an individual plant can have from one to hundreds of stems growing in well-defined patches [21]. The complex flower morphology consists of three outer tepals that fall downwards (“falls”) and three inner upright tepals (“standards”). At the base of each fall tepal, there is a dark mark, a cluster of specialized black cells that appear to be black regardless of the flower’s color, which will be referred to herein as the “black patch”. Behind this black patch on the fall tepal, a petaloid style forms a tunnel-shaped space together with the base of the fall. Each flower has three black patches and three tunnels (Figure 1).

Royal irises are self-incompatible and rely on pollinators for their reproduction [21]. A bee transporting pollen and sheltering in at least one tunnel might perform fertilization and fruit development [21]. The seeds are dispersed by ants and are carried up to 60 m away from the plant [39]. These flowers do not produce any nectar, but they offer shelter as a reward to *Eucera* male bees that use the tunnels of the flowers as shelters overnight [21]. Bees that shelter in flower tunnels emerge earlier in the morning than bees sheltering on the bare ground [40]. This observation is associated with the increased temperature within the tunnels compared with the ambient air temperature in the first 60–90 min after sunrise [40]. Thus, a heat reward has been suggested to be associated with the night-sheltering reward system.

Previous studies have shown a positive-mediated selection on flower size in *Iris atropurpurea* [38]. The high correlations between rainfall and flower size, but not with black patch size in *Iris petrana*, suggest that flower size might be selected both by water availability and pollinators, while the size of the black patch might be selected by pollinators only [22]. However, whether the size of the tunnel is related to the flower size and black patch size, and its covariation with fitness, remain unclear.

Here, we tested whether flower and black patch sizes act as honest signals for a shelter size (tunnel volume) reward in Royal irises. Larger flowers might increase the plant’s attractiveness to pollinators from a distance, while at closer range, the black patch might signal the location of the reward (i.e., entrance to the shelter). If flower and black patch sizes are honest signals for the shelter reward, they are predicted to be in close correlation with the tunnel size, and this correlation would be under selection. This prediction assumes that more bees could shelter in larger tunnels, increasing the likelihood of a pollination event and the probability of a flower becoming a fruit and producing seeds. We measured the flowers of seven species of Royal irises from the collection of the Tel Aviv University Botanical Garden (TAUBG) to test the relationship between flower size, black patch size, and tunnel volume. In addition, we tested the effects of flower size, black patch size, and tunnel size, and their correlations on fitness, in three natural populations of *Iris atropurpurea* and *Iris petrana* that differ in terms of their flower sizes.

## 2. Results

Flower traits including flower size, black patch size, and tunnel volume (Figure 1A–C) were measured in seven species of Royal irises (Figure 1D) from a controlled environment at the Tel Aviv University Botanical Garden (TAUBG). Additionally, the flower traits were measured in three natural populations of two species: *Iris petrana* in Yeruham (YER), and *Iris atropurpurea* in Netanya (NET) and Yavne-Kur (KUR). Hereafter, these populations are referred to as YER, NET, and KUR, respectively.

### 2.1. Controlled Environment (TAUBG)

**Figure 2:**
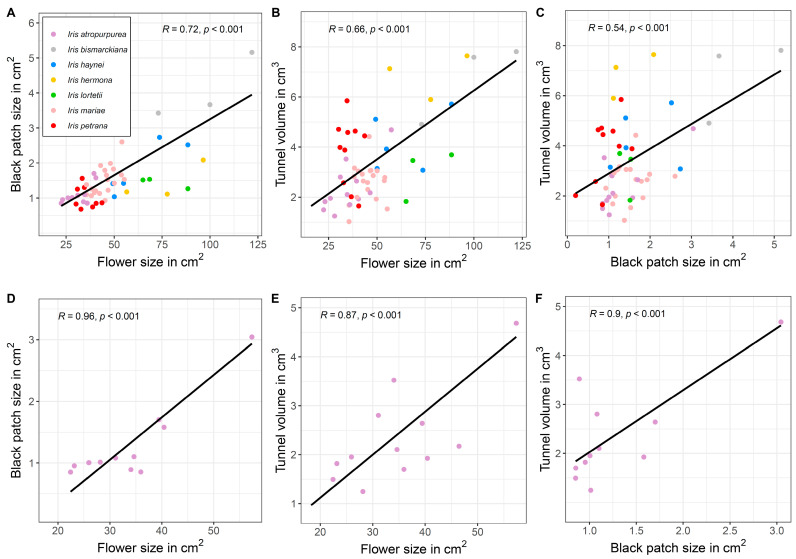
Flower size, black patch size, and tunnel volume in seven species of Royal irises from the TAUBG collection. When all species were analyzed together, all flower traits exhibited positive relationships (**A**–**C**). When species were analyzed separately, significant correlations of all flower traits were only found in *Iris atropurpurea* (**D**–**F**).

Flower size at TAUBG collection did not differ between 2021 and 2022 (LM, F_1, 163_ = 1.64, *p* = 0.20, Supplementary Figure S1). When all species were analyzed together, we found positive correlations between all three flower traits. Flower size was positively correlated with black patch size (Pearson´s r = 0.72, *p* < 0.001, Figure 2A) and tunnel volume (Pearson´s r = 0.66, *p* < 0.001, Figure 2B). Black patch size was also positively correlated with tunnel volume (Pearson´s r = 0.54, *p* < 0.001, Figure 2C). When the species were analyzed separately, significant positive relationships for all combinations were found in *Iris atropurpurea* only (Figure 2D–F), except for *I. mariae*, which showed a positive significant relationship between black patch size and flower size (Supplementary Figure S2).

### 2.2. Natural Populations

**Figure 3:**
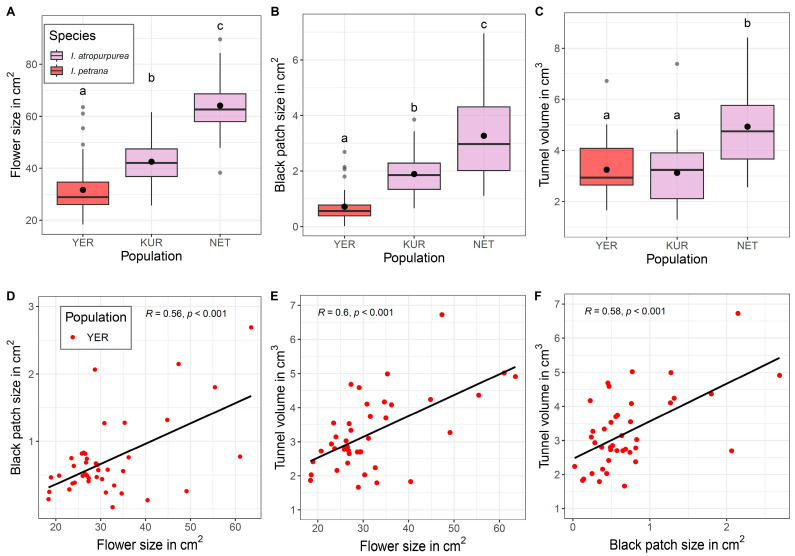
Flower size, black patch size, and tunnel volume of *Iris atropurpurea* (KUR and NET) and *Iris petrana* (YER). Flower size (**A**), black patch size (**B**), and tunnel volume (**C**) varied between species and populations. When populations were analyzed separately, there was only a positive relationship between tunnel, black patch size, and flower size in the *Iris petrana* YER population (**D**–**F**). Letters in (**A**–**C**) indicate significant differences between populations; black circles indicate mean values.

In the wild, flower size differed among populations, with larger flowers found in NET, followed by KUR and YER (LM, F_2, 116_ = 87.51, *p* < 0.001, Figure 3A). A similar tendency was found in black patch size (LM, F_2, 116_ = 89.70, *p* < 0.001, Figure 3B). Tunnel volume was the largest in NET but similar in YER and KUR (LM, F_2, 116_ = 20.13, *p* < 0.001, Figure 3C). Significant positive correlations for all flower traits were found in YER (Figure 3D–F), as well as a significant positive relationship between black patch size and tunnel volume in the *I. atropurpurea* NET population (Supplementary Figure S3).

### 2.3. Fitness from Natural Populations

From a total of 93 flowers marked, 51 developed into fruits, but not all of them bore seeds (Table 1). In the YER population, 75% of fruits had seeds, followed by the KUR population with 62%, and the NET population with 40%. The mean number of seeds per fruit did not differ significantly between YER and KUR (~18 seeds/fruit); the NET population had an average of 5 seeds/fruit (Table 1, Supplementary Figure S4).

**Figure 4:**
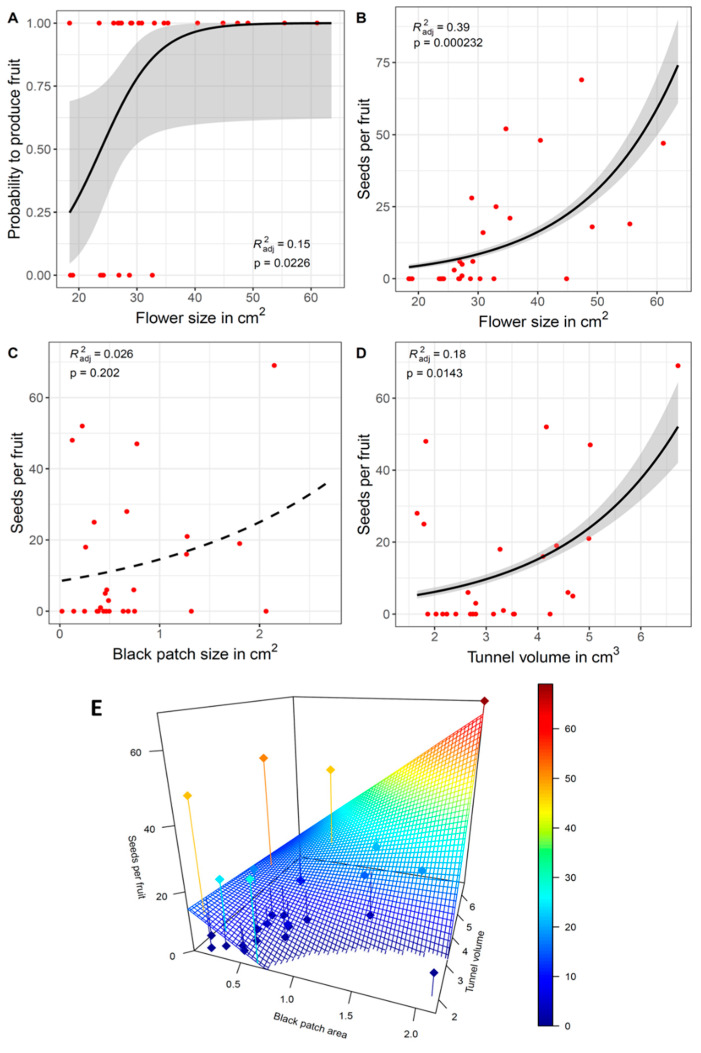
Effect of flower size, black patch size, and tunnel volume on fitness in the YER population. Flower size increased the probability of producing fruits (**A**) and the number of seeds per fruit (**B**). Black patch size alone did not affect the seed set (**C**), while tunnel volume (**D**) and black patch size × tunnel volume interactions (**E**) had a positive effect on the seed set. Dashed lines indicate no significance.

Fruit set, measured as the probability of a flower to become a fruit, significantly increased with flower size and tunnel volume in the *Iris petrana* YER population (Table 2, Figure 4A). Similarly, seed set, measured as the number of seeds per fruit, significantly increased with the flower size and tunnel volume in this population (Table 2, Figure 4B,D). The size of the black patch did not affect the seed set, but its interaction with tunnel volume did (Table 2, Figure 4C,E). No relationships between fruit and seed set with flower size, black patch size, tunnel volume, or their interactions were found in the KUR population (Table 2). Fruit set did not show any effect on single flower traits in the NET population, but their interactions had a significant effect on seed set (Table 2, Supplementary Figure S5).

## 3. Discussion

Honest signaling in plant–pollinator systems is key in the coevolution of flowers and pollinating animals. Flower size and coloration signals have scarcely been studied in this context. In this study, we tested whether two visual signals, flower size and black patch size, act as honest signals in the Royal irises. The flowers of Royal irises offer a tunnel-shaped shelter as a reward, where bees shelter during the night. Here, we tested the relationship between flower size and black patch size with tunnel volume (as an indicator for the extent of the shelter reward) and the possible selection acting on these advertisements and the reward.

We found evidence for honest signaling in a controlled environment, expressed as a positive relationship between either flower size or black patch size and tunnel volume (Figure 2). However, in the natural populations, this positive relationship was found only in *Iris petrana* (Figure 3). In addition, the interaction between traits, i.e., the correlation between flower/patch size and tunnel size, increased fitness in *I. petrana* YER and *I. atropurpurea* NET populations (Figure 4, Table 2). This suggests a positive selection on the honesty of the signal, and a correlation between advertisement and reward in these populations.

### 3.1. Honest Signals of Flower and Black Patch Are Species- and Population-Specific

Although we found a positive relationship between the visual signals (i.e., flower size and black patch size) and the reward (i.e., tunnel volume), this honest signaling was not present in all species and/or populations, evident only in *Iris atropurpurea* in the controlled environment, and in *Iris petrana* YER population in the wild, as well as in the *Iris atropurpurea* NET population. These results suggest that flower size and black patch size are honest signals for the size of the reward, but this honest signaling varies across species and populations.

Population-specific selection on flower traits mediated by pollinators was previously documented in other species (e.g., [41,42,43,44,45]). In Royal irises, pollinator-mediated selection on flower size was shown in *Iris atropurpurea*, but not in *Iris haynei* [38]; our results suggest that selection mediated by pollinators varies between the two populations of *Iris atropurpurea*. *Iris petrana* did not show any significant correlation under controlled conditions, but the natural population of YER did, as well as a clear positive effect of flower size and tunnel volume on fitness. These results highlight the differences among populations, but whether these relationships are also present in other populations of *I. petrana* remains to be tested. More important than the effect of single flower traits (i.e., flower size and black patch size) on fitness was the effect of their interaction with tunnel volume. All interactions were significant in the NET population, although in the YER population, only black patch size × tunnel volume had an effect on fitness (Table 2). These results suggest selection of the honest signal, which varies between populations as well.

### 3.2. Are Larger Shelters Better?

In Royal irises, shelter is the reward [20,21]. Larger tunnels might offer more space for bees to shelter. *Eucera* bees tend to sleep in aggregations [46,47,48], which help them maintain higher body temperature [49], or as a dilution effect against predators [48]. Previous observations in *I. atropurpurea* showed an average of 2 male bees sheltering in a single tunnel (mode = 1), and an exponential decrease in the frequency of number of bees, up to a single case of 22 bees in one tunnel (Y. Sapir unpublished). Therefore, aggregations (at least 2 bees) seem to be common, likely increasing pollen deposition and pollen import.

Pollination success highly depends on the pollinator visitation rate and pollinator preference [50,51,52]. Interactions between flower/patch size and tunnel size, and increased fitness in *I. petrana* YER and *I. atropurpurea* NET populations, suggest that larger flowers and patches might be preferred by pollinators in these populations [22,38]. However, pollen limitation was previously identified in the NET population [38], which might explain the high fraction (60%) of fruits with zero seeds observed in this study. In the *I. atropurpurea* KUR population, a high proportion of fruits with seeds (75%) and a high number of seeds per fruit (~18, similar to YER; Table 1, Supplementary Figure S4), indicate that pollination events do occur there. However, lack of evidence for an honest signal or its effect on fitness in this population (Table 2) suggests that pollinators might not be driven by flower or black patch size. More studies including direct observations on pollinators’ behavior are needed.

Larger tunnels may provide better shelters as a reward. Previously, temperature increase within the tunnels after sunrise was argued to be the reward to night-sheltering pollinators [40]. However, the relationship between tunnel size and this heat reward remains unclear. We hypothesize that larger tunnels provide a better microclimate that heats up faster than smaller tunnels in the morning. Nonetheless, this hypothesis is still not resolved.

### 3.3. Abiotic Factors Can Affect the Selection of Flower Size

Resource availability is an important limiting factor on flower traits, including flower size [53]. In Royal irises, flower size relates to the north–south aridity gradient of Israel, decreasing towards the desert, as an adaptation to drought [34]. In arid habitats where water and nutrients are scarce, large flowers can be very costly to produce while small ones are favored [54,55], causing changes in plant–pollinator interactions [56,57].

The decrease in flower size of the seven Royal irises measured under controlled conditions matches their natural distribution from north to south (Figure 1, Supplementary Figure S6A), following the aridity gradient (for the natural occurrence of the species, see Figure 1 in Ref. [58]). Black patch size and tunnel volume did not exhibit such a decrease, and seem to be less variable among species (Supplementary Figure S6B,C), suggesting that the climate gradient does not affect these traits as much as it affects flower size.

The natural occurrence of *Iris petrana* is located in the Negev desert, an arid environment with low water availability. In the YER population, flower size was found to highly depend on rainfall over the years, while black patch size remained constant [22], suggesting that flower size is a very plastic trait highly dependent on water availability and costly for the plant to produce. In addition, we found a direct positive effect of flower size on fitness in this population, which suggests that pollinators select larger flowers with larger tunnels. This might cause a selection conflict where the climate might select for smaller flowers, while pollinators select for larger flowers.

In the *Iris atropurpurea* NET population, no direct effect of flower size on fitness was found, but interactions of flower size and black patch size with tunnel volume increased the seed set (Table 2). These results suggest that both traits together might be important and likely selected by pollinators. Indeed, pollinator-mediated selection on flower size was previously found in this population [38]. Whether pollinators still prefer larger flowers/patches occurring in populations under more favorable conditions (i.e., populations in the north) remains unclear. More studies to test the effect of water availability on reproductive success and the effect of pollinators are needed [59].

### 3.4. Is There an Indirect Selection of Black Patch Size?

We found a positive relationship between flower and black patch size, and between black patch size and tunnel volume, both in controlled conditions and in wild populations (Figure 2 and Figure 3). However, in the natural populations, black patch size did not affect fruit and seed sets (Table 1). In the *Iris petrana* YER population, larger flowers with larger tunnels significantly increased fitness, while in *Iris atropurpurea*, the interactions of flower size × black patch size and black patch size × tunnel volume increased fitness, although this effect was population-specific (in the NET population only). There was no direct effect of black patch size on the fitness component of either species or populations, but the significant effect of tunnel volume × black patch size interaction on fitness suggests a complex synergistic effect of the signal and the reward, which may hint for an indirect selection on this signal.

While the size of the black patch might not be under selection per se, the selection may act indirectly through the black patch in gaining heat and transferring the energy to the tunnel, playing a role on the heat reward (Y. Sapir and R. Heliczer, unpublished). An ongoing study is comparing temperature increase within the tunnels in these natural populations and the role of the black patch as an underlying mechanism of flower heating (Lozada-Gobilard et al., in preparation).

## 4. Materials and Methods

### 4.1. Flower Size, Black Patch Size, and Tunnel Volume Measurements

To determine the size of the flower, we measured the length and the width using calipers and calculated a flower size by multiplying them together (Figure 1A). Black patch area was estimated from digital photographs using ImageJ [60]. Each black patch was carefully, manually encircled, and its area was calculated using the standardized measuring protocol implemented in ImageJ (Figure 1B). Tunnel volume was calculated by multiplying the length and the width of the tunnel entrance with the depth of the tunnel (Figure 1C). For black patch size and tunnel volume, one tunnel and fall tepal out of the three were selected randomly. Flower and black patch size are presented in cm^2^, while the tunnel volume is in cm^3^.

### 4.2. Sampling of Plant Material

The flower measurements were collected from the Royal irises collection, and maintained in a nethouse at the Tel Aviv University Botanical Garden (TAUBG; 32°06′ N, 34°48′ E), a controlled isolated environment, permissible to light but impermissible to pollinators (Supplementary Figure S7). The collection was established in 2008–2009 by transplanting rhizomes from natural populations throughout Israel. Rhizomes were transplanted to individual bags with garden soil and tuff (50:50) and watered regularly (~2 L per week) with an automatic irrigation system. Rhizomes of the plants are replanted in new soil approximately every five years.

Seven species of Royal irises were measured, namely, *Iris bismarckiana*, *I. hermona*, *I. lortetii*, *I haynei*, *I. mariae*, *I. atropurpurea*, and *I. petrana*, representing species endemic or sub-endemic to Israel (Figure 1D). We measured 59 flowers in total: *I. petrana* (n = 10), *I. atropurpurea* (n = 16), *I. mariae* (n = 19), *I. haynei* (n = 5), *I. lortetii* (n = 3), *I. hermona* (n = 3), and *I. bismarckiana* (n = 3). In their natural environment, these species are distributed along the north–south aridity gradient of Israel [34] and are eco-geographically isolated with different levels of pre- and post-zygotic reproductive barriers [58,61]. For an overview of the natural distributions of these species in Israel and Palestine, see Figure 1 in Ref. [58].

Additionally, flower traits were measured in three natural populations of two species: *Iris petrana* in Yeruham (YER), and *Iris atropurpurea* in Netanya (NET) and Yavne-Kur (KUR). Sample sizes: YER (n = 41), KUR (n = 48), and NET (n = 30). Both KUR and NET are located in the Mediterranean coastal region of Israel. The NET population is at the Netanya Iris reserve, located 26 km north of Tel Aviv (32°170 N, 34°500 E, altitude 37 m), and KUR is 15 km south of Tel Aviv (31°53′25.4 N, 34°42′35.60 E, altitude 15 m). The YER population is in the Yeruham Iris Nature Reserve, located in the arid region of Israel (31°01′14.46 N, 34°58′21.4 E, altitude 549 m). These three populations occur along a latitudinal gradient from north to south, receiving 600, 500, and 200 mm of mean annual precipitation, respectively. The average temperature from February to April in NET is 18–23 °C, 19–25 °C in KUR, and 18–26 °C in YER. The average temperature from February to April in NET varies from 18 to 23 °C, from 19 to 25 °C in KUR, and from 18 to 26 °C in YER.

Data from both TAUBG and natural populations were collected during the Royal irises flowering season, between February and April 2022. *Iris atropurpurea* exhibits wide within-species variation in flower size; thus, to account for most of the variation, we selected two extreme populations with large (NET) and small (KUR) flowers, while *Iris petrana* corresponds to the smallest range among all species (see the average flower sizes per population in Supplementary Table S1). Collection of data in the wild for these two species was possible since their flowering times do not overlap (*I. atropurpurea* = January until mid-March; *I. petrana* = March to April). Populations of larger flowers such as *I bismarckiana* or *I hermona* flower simultaneously with *I. petrana*, making their data collection logistically difficult.

It was previously shown that flower size highly depends on water availability (i.e., rainfall) [22]. To ensure that flower size did not change under the controlled conditions of the TAUBG collection, we measured flower sizes in two consecutive years (2021 and 2022) and tested whether there were differences between the two years in flower size. Tunnel and black patch size data were collected in 2022 only. Since the environmental conditions in the natural populations vary, we tested the relationships between flower, black patch size, and tunnel volume by population.

### 4.3. Fitness Estimates in Natural Populations

In the three natural populations of NET, KUR, and YER, we randomly marked flowers that were opened for at least 5 days and bagged them to preserve the fruits and seeds for later collection. In total, 119 flowers were measured for flower traits (YER = 41, KUR = 48, and NET = 30), and 93 were marked for fruit development. In YER, 31/41 flowers were marked, 28 were later recovered, and of these, 20 (71%) flowers developed into fruits. In KUR, 35/48 flowers were marked, 23 recovered, and 16 (69%) developed into fruits. In NET, 27/30 were marked, 19 recovered, and 15 (79%) developed into fruits (Table 1). Since these species are self-incompatible, at least one efficient visit to the tunnel of an *Eucera* bee is needed for fertilization. Moreover, because all three stigma lobes are merged into one style, pollen deposition on a single stigma is sufficient to fertilize seeds in all three carpels of the ovary (Y. Sapir, unpublished). We recorded whether the marked flowers developed into a fruit (Yes = 1, No = 0). In YER, a total of 31 flowers were marked, corresponding to 15 individuals. In NET, 57 flowers were bagged, corresponding to 24 individuals, while in KUR, 35 flowers corresponding to 13 individuals were marked. Due to a high variation in flower size within genotype [22] and some cases with a lack of clear separation between individuals (S. Lozada-Gobilard pers. obs.), each flower was analyzed independently; hence, the analysis considers the ecological interaction of the single flower, rather than the fitness of the genotype (individual plant). From the collected fruits, we recorded whether they developed seeds and counted the number of seeds per fruit. Flowers that did not set fruits, and fruits that did not contain seeds, were recorded as zero seeds.

### 4.4. Statistical Analyses

The TAUBG and natural populations datasets were analyzed separately. Flower size and tunnel volume from the TAUBG dataset followed a normal distribution, while the black patch size was log-transformed to improve normality. All three variables were log-transformed from the dataset of the natural population to improve normality. To evaluate whether flower size changed in two years at the TAUBG, we applied a linear model using the “*lm*” and “*Anova*” functions. To develop a general overview about the relationships between flower size, black patch size, and tunnel volume in Royal irises, we applied Pearson’s correlations, including all species in the TAUBG dataset (N = 59), and separately by species for those with N > 5 (i.e., *I. atropurpurea*, *I. petrana*, and *I. mariae*). Only the flower size measurements from TAUBG corresponding to 2022 were used.

To compare populations from the natural populations dataset, we applied a linear model using “*lm*” from the “stats” package and the “*Anova*” function from the “car” package, and performed pairwise comparison using Tukey post hoc tests with the “*TukeyHSD*” function from the “stats” package. Correlations within populations (KUR, NET, and YER) were analyzed separately using Pearson’s tests. To test whether flower size, black patch size, or tunnel volume influenced fruit set, we applied a generalized linear model (GLM) with a binomial family distribution. Finally, to test the effect on seed set between populations, we applied log-linear regression using GLM with a Poisson family distribution suitable for counting data. All statistical tests were performed using R software version 4.2.2 [62].

## 5. Conclusions

In this study, we tested the hypothesis that flower size and black patch size are honest signals for the shelter reward (tunnel volume) in Royal irises. Our results showed that flower size and black patch size could act as an honest signal, where large flowers/patches indicate larger tunnels (where pollinators shelter), increasing the probability of fruits and seeds. Under controlled conditions, evidence of honest signaling was found in an entire group of Royal irises, but only in *Iris atropurpurea*. However, in the wild, this positive relationship was only found in YER *Iris petrana* and NET *Iris atropurpurea* populations. These results suggest that the positive relationship between flower/patch size and tunnel volume might be a common trait in Royal irises, but its effect on fitness might be species- or population-specific. In addition, flower size showed a direct positive effect on fitness in the YER *I. petrana* population; correlation between flower/patch size and tunnel size (i.e., interactions between traits) increased fitness in YER *I. petrana* and NET *I. atropurpurea* populations, suggesting a positive selection on the honesty of the signal. These results suggest that flower size might act as an honest signal for larger tunnels putatively attracting pollinators from a distance, whereas the size of the black patch at closer range might not be as important as the size of the flower. More studies are needed to evaluate pollinator preferences and selection on flower size, black patch size, and tunnel size in more species of Royal irises with an extended size range. Understanding how flower traits attract pollinators is essential for plant fitness, in particular for those that fully depend on pollinators to reproduce. Changes in flower traits due to various factors, including pollinators, directly affect plant reproduction success, which is crucial for conservation purposes, especially of endemic plant species such as the Royal irises.

## Figures and Tables

**Table 1:** Summary of fitness measurements and sample sizes in the natural populations. Populations KUR and NET correspond to *Iris atropurpurea*, while YER corresponds to *I. petrana*.

Pop	N Plants	Flowers Measured for Traits	Flowers Marked for Fitness	Flowers That Became Fruits (%)	Fruits with Seeds (%)	Seeds per Fruit (Mean ± SD)
KUR	15	48	35	16 (46%)	10 (62%)	17.6 ± 2.5
NET	13	30	27	15 (56%)	6 (40%)	4.6 ± 1.3
YER	23	41	31	20 (65%)	15 (75%)	18.2 ± 3.2

**Table 2:** Effects of flower size, black patch size, and tunnel volume on the fruit set and seed set in three populations of Royal irises. Significant effects are in bold. Asterisks represent significance levels: ** *p <* 0.01, * *p <* 0.05, (*) shows values that are marginally significant ~0.05.

Species	Pop	Predictors		Fruit Set		Seed Set	
			Df	F Value	*p* Value	F Value	*p* Value
*I. atropurpurea*	KUR	Flower size	1	0.16	0.686	0.33	0.568
		Black patch size	1	0.00	0.980	0.07	0.786
		Tunnel volume	1	0.10	0.752	0.45	0.511
		Flower size × Black patch size	1	0.66	0.428	0.62	0.441
		Flower size × Tunnel volume	1	0.86	0.368	0.55	0.465
		Black patch size × Tunnel volume	1	0.57	0.458	0.13	0.715
	NET	Flower size	1	1.15	0.301	1.13	0.030
		Black patch size	1	1.49	0.240	2.26	0.154
		Tunnel volume	1	2.24	0.156	0.07	0.793
		Flower size × Black patch size	1	1.08	0.314	**10.6**	<0.01 **
		Flower size × Tunnel volume	1	1.80	0.200	**7.87**	<0.05 *
		Black patch size × Tunnel volume	1	**3.71**	0.07 (*)	**5.40**	<0.08 (*)
*I. petrana*	YER	Flower size	1	**8.62**	<0.01 **	**8.48**	<0.01 **
		Black patch size	1	0.30	0.586	0.23	0.636
		Tunnel volume	1	**3.41**	0.07 (*)	**5.01**	<0.05 *
		Flower size × Black patch size	1	1.78	0.193	0.40	0.532
		Flower size × Tunnel volume	1	0.64	0.429	0.54	0.470
		Black patch size × Tunnel volume	1	1.17	0.289	**5.12**	<0.05 *

## Data Availability

Data and R script code are available on Figshare at https://doi.org/10.6084/m9.figshare.23596698 (accessed on 16 October 2023).

## Supplementary Materials

The following supporting information can be downloaded at: https://www.mdpi.com/article/10.3390/plants12162978/s1, Figure S1. Comparison between 2021 and 2022 of flower size measurements in the TAUBG collection. Figure S2. Relationship between flower size and black patch size per species n > 5 from the TAUBG. Figure S3. Relationship between flower size, black patch size, and tunnel volume per population. Figure S4. Proportion of fruits that developed seeds and the mean number of seeds per fruit per population. Figure S5. Single and interactive effects of flower size, black patch size, and tunnel volume on seed set in the NET population. Figure S6. Flower traits measured in seven species of Royal irises under controlled conditions of the TAUBG. Figure S7. Royal irises collection at the Tel Aviv University Botanical Garden. Table S1. Sampling overview of individuals from the TAUBG and natural populations.
