# Supplementary material for: Flower Size as an Honest Signal in Royal Irises (Iris Section Oncocyclus, Iridaceae)
Source: Plants (Basel). 2023 Aug 18;12(16):2978. doi: 10.3390/plants12162978 (PMC10459770; doi:10.3390/plants12162978)
Supplement: Supplementary file 1 [file plants-12-02978-s001.zip › plants-2503743-supplementary.pdf]

Flower size as an honest signal in Royal Irises (*Iris* section *Oncocyclus*, Iridaceae)

Lozada-Gobilard et al

SUPPLEMENTARY MATERIALS

LIST OF FIGURES

Figure S1. Comparison between 2021 and 2021 of flower size measurements in the TAUBG collection.

Figure S2. Relationship between flower size and black patch size per species n>5 from TAUBG.

Figure S3. Relationship between flower size, black patch size and tunnel volume per population.

Figure S4. Proportion of fruits that developed seeds and mean number of seeds per fruit per population.

Figure S5. Single and interactive effects of flower, black patch size and tunnel volume on seed set in NET population.

Figure S6. Flower traits measured in seven species of Royal irises under controlled conditions of TAUBG.

Figure S7. Royal irises collection at Tel Aviv University Botanical Garden.

LIST OF TABLES

Table S1. Sampling overview of individuals from TAUBG and natural populations.

FIGURES

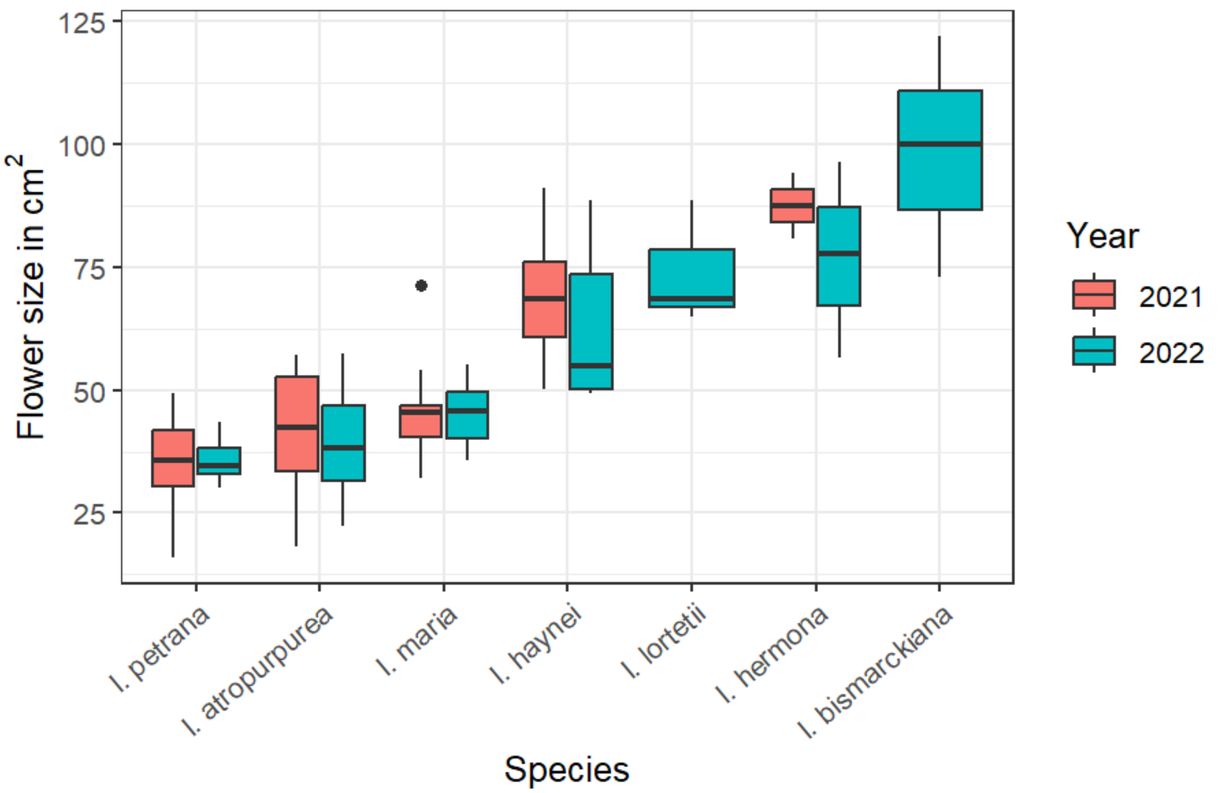

Figure S1. Comparison between 2021 and 2022 of flower size measurements in the TAUBG collection. There was a significant effect of species on flower size but year had no effect. Note that not all species flowered in 2021. Sample sizes: *I. petrana* (n_{total}=34; n_{2021}=24; n_{2022}=10), *I. atropurpurea* (n_{total}=64; n_{2021}=42; n_{2022}=22), *I. mariae* (n_{total}=40; n_{2021}=20; n_{2022}=20), *I. haynei* (n_{total}=16; n_{2021}=11; n_{2022}=5), *I. lortetii* (n_{total}=3; n_{2021}=0; n_{2022}=3), *I. hermona* (n_{total}=5; n_{2021}=2; n_{2022}=3), (n=5), *I. bismarckiana* (n_{total}=3; n_{2021}=0; n_{2022}=3).

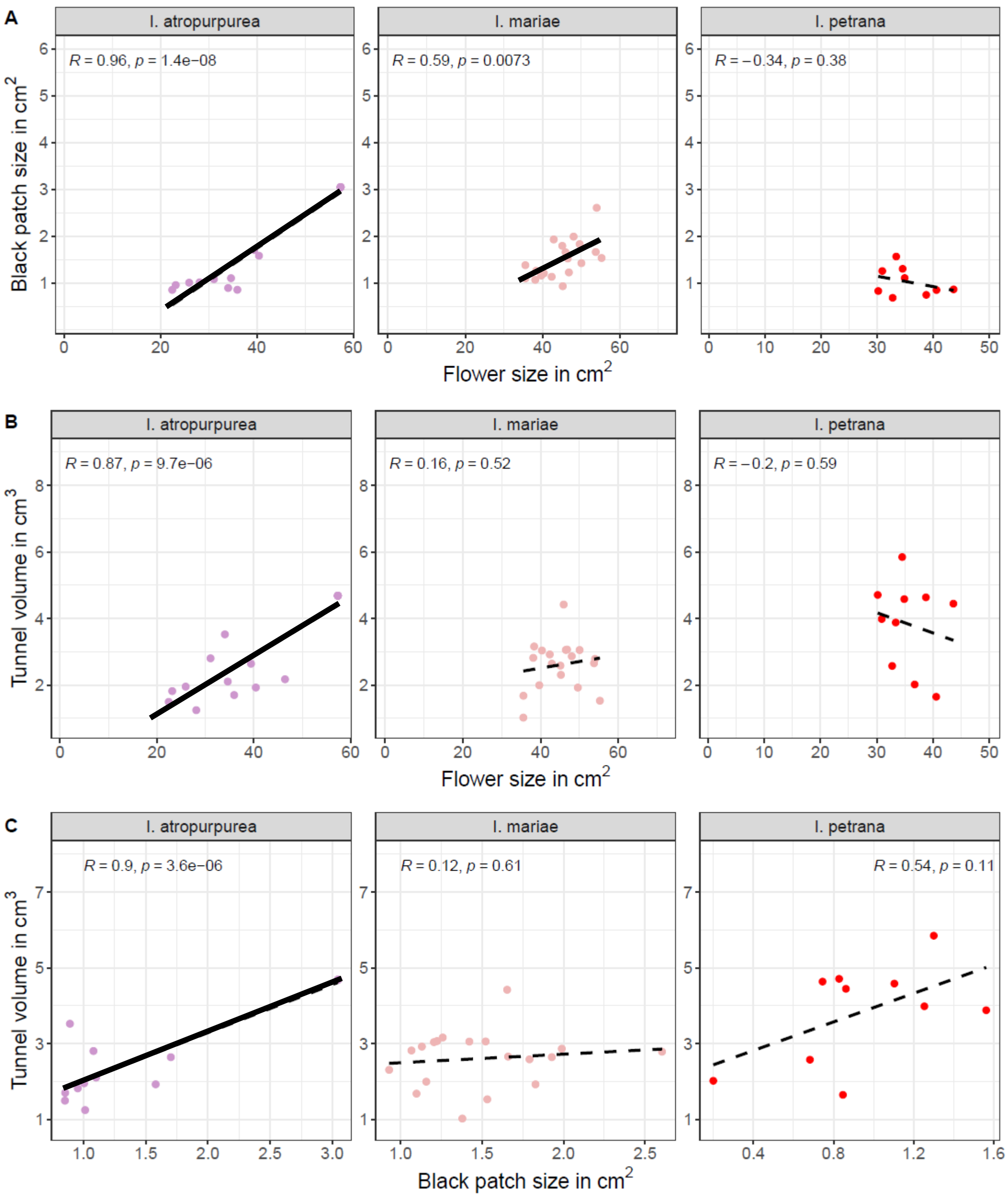

Figure S2. Relationship between flower size and black patch size per species n > 5 from TAUBG. Flower size showed a general positive tendency in most species, but only significant in *Iris atropurpurea* (A-C) and between flower size and black patch size in *I. mariae* (A). Sample sizes: *I. petrana* (n=10), *I. atropurpurea* (n=16), *I. mariae* (n=19). Dashed lines indicate no significance. Flower traits correspond to 2022 since black patch size and tunnel volume were measured in 2022 only.

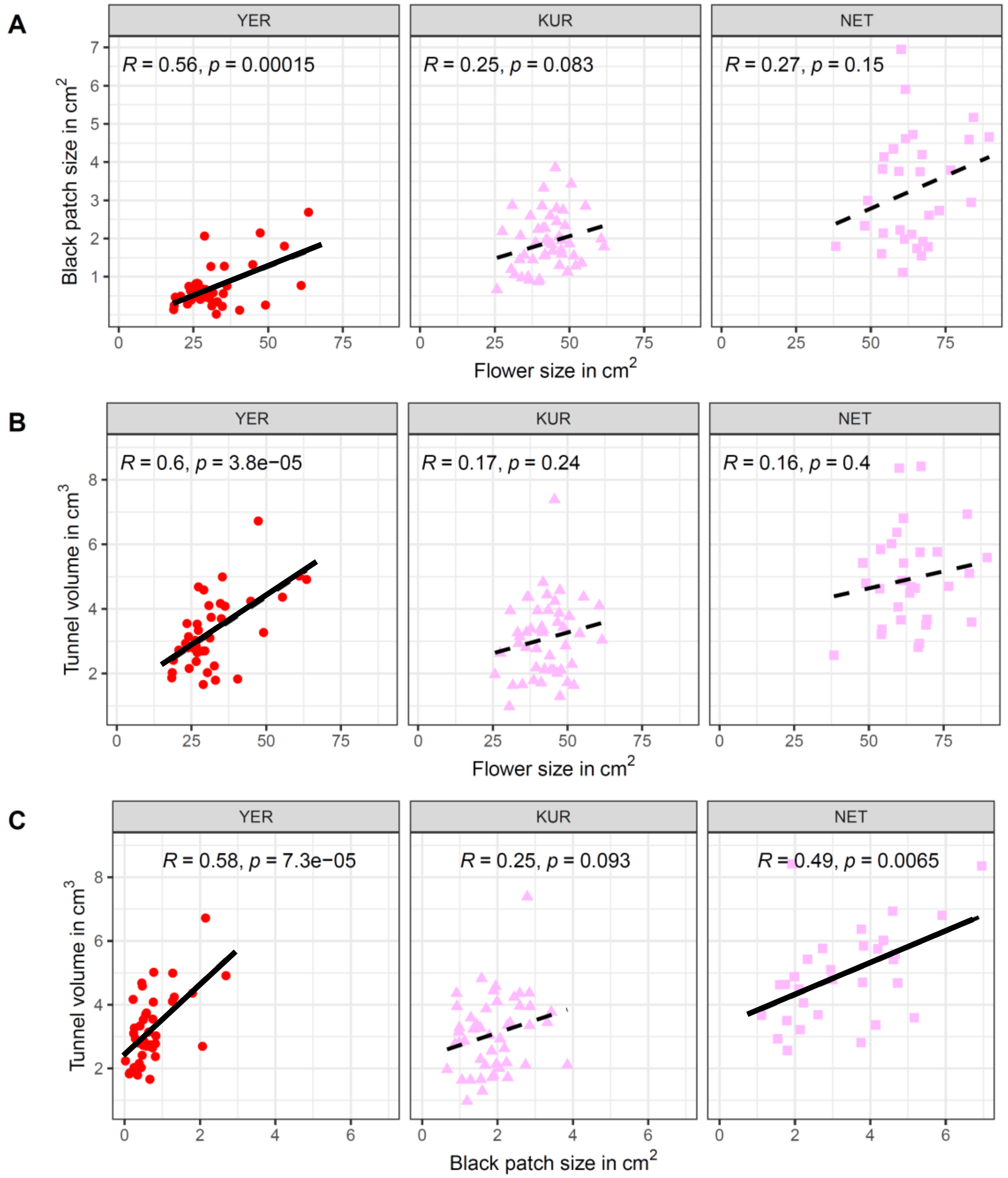

Figure S3. Relationship between flower size, black patch size and tunnel volume per population. There was a significant positive relationship among all traits in YER *I. petrana* population (**A-C**). In NET *I. atropurpurea* population, a significant relationship between tunnel volume and black patch size was found (**C**). N samples YER=41, KUR=48, NET=30. Dashed lines indicate no significance. Flower traits correspond to 2022 since black patch size and tunnel volume were measured in 2022 only.

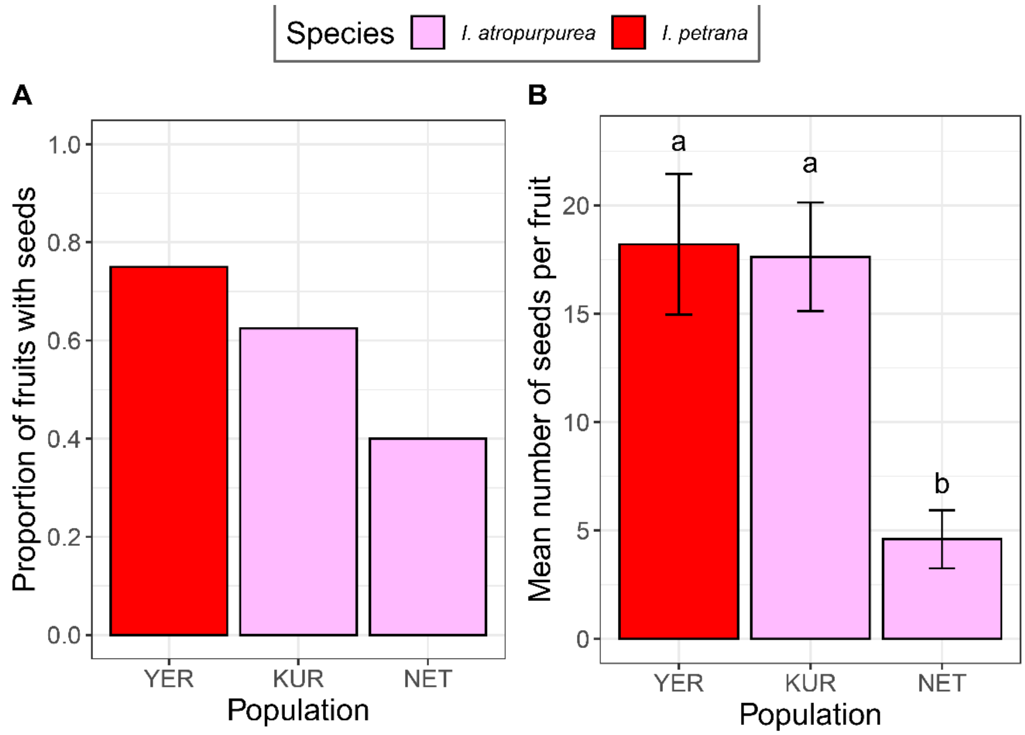

Figure S4. Proportion of fruits that developed seeds and mean number of seeds per fruit per population. The highest number of fruits with seeds (**A**) and number of seeds per fruit (**B**) was found in YER, followed by KUR and NET populations. Letters in B indicate significant differences among populations.

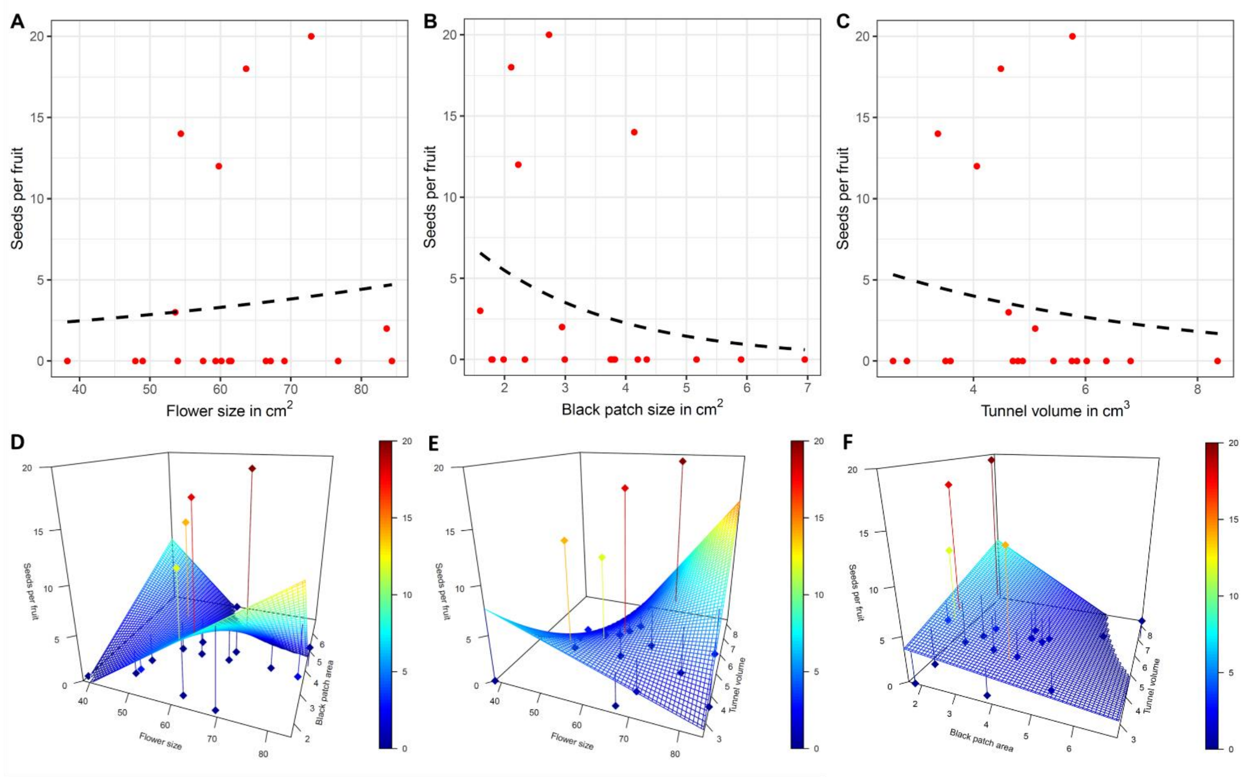

Figure S5. Single and interactive effects of flower, black patch size and tunnel volume on seed set in the NET population. Single effects of flower size (**A**), black patch size (**B**) and tunnel volume (**C**) did not have a significant effect on seed set, while all the interactions did (**D-F**). Dashed lines indicate no significance.

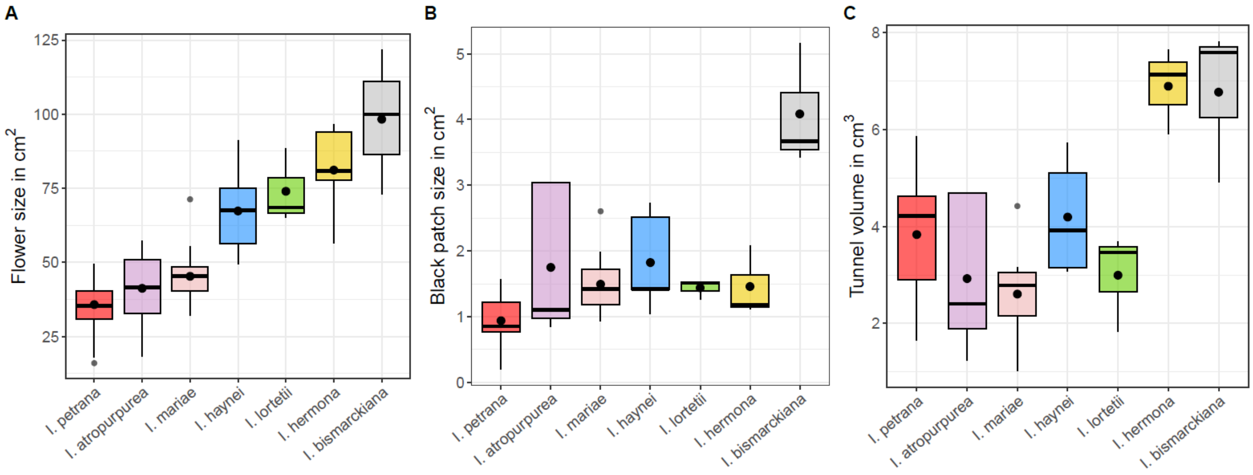

Figure S6. Flower traits measured in seven species of Royal irises under controlled conditions of TAUBG. Flower size decrease matches the north-south aridity gradient of Israel (**A**), while the black patch size (**B**) and tunnel volume (**C**) are less variable forming two distinct groups. Points inside boxplots indicate mean values. Sample sizes: *I. petrana* (n=10), *I. atropurpurea* (n=16), *I. mariae* (n=19), *I. haynei* (n=5), *I. lortetii* (n=3), *I. hermona* (n=3), *I. bismarckiana* (n=3).

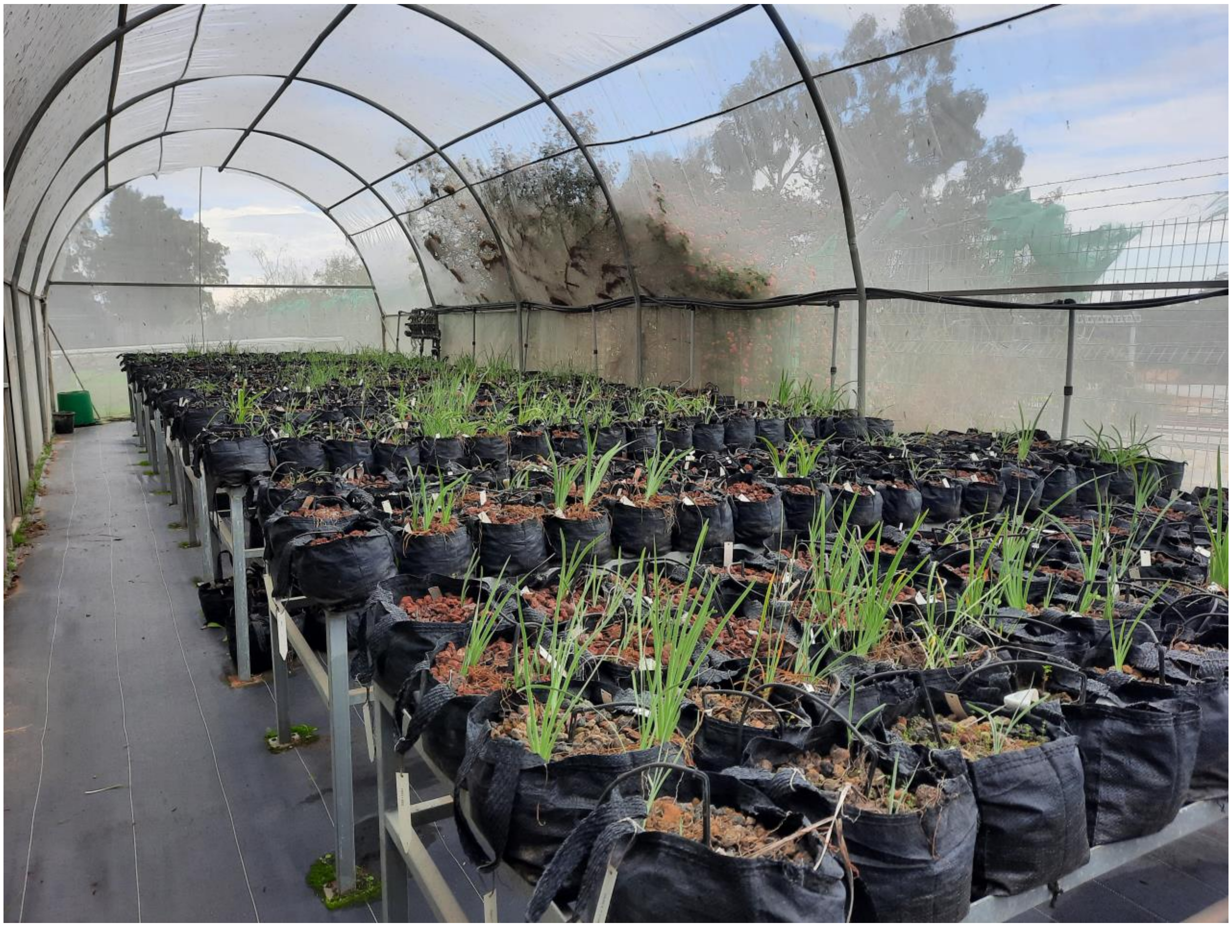

Figure S7. Royal irises collection at Tel Aviv University Botanical Garden (TAUBG). Each individual bag contains 2-3 rhizomes from the same individual transplanted from natural populations. A total of seven species: *Iris petrana, I. atropurpurea*, *I. mariae*, *I. haynei, I. lortetii,* *I. hermona* and *I. bismarckiana* from different populations are grown in the collection.

Table S1. Sampling overview of individuals from TAUBG and natural populations. Flower size is averaged between 2021 and 2022. In parenthesis the number of plants that correspond to the number (n) of flowers collected.

Species	Population	Flower size [cm²]				Black patch size [cm²]				Tunnel volume [cm³]			
		n	mean	±	SE	n	mean	±	SE	n	mean	±	SE
**Controlled environment TAUBG dataset**													
Iris atropurpurea	KDM	1 (1)	40.43	±	--	1 (1)	1.58	±	--	1	1.92	±	--
	KUR	5 (2)	27.30	±	2.64	3 (2)	0.96	±	0.07	3	2.04	±	0.39
	NET	15 (2)	53.66	±	0.81	5 (1)	3.04	±	0.001	5	4.68	±	0.00
	NRG	2 (2)	30.03	±	11.78	--	--	-	--	--	--	-	--
	NSZ	13 (6)	40.51	±	2.12	--	--	-	--	--	--	-	--
	NTA	9 (7)	34.02	±	3.24	3 (3)	1.24	±	0.23	3	1.95	±	0.40
	PAL	3 (3)	33.75	±	1.79	1 (1)	0.89	±	--	1	3.52	±	--
	SHF	4 (4)	55.38	±	0.58	--	--	-	--	--	--	-	--
	TLV	12 (4)	36.78	±	1.91	3 (2)	0.98	±	0.10	3	1.99	±	0.15
Iris bismarckiana	SMS	3 (3)	98.28	±	14.13	3 (3)	4.09	±	0.54	3	6.77	±	0.93
Iris haynei	HMD	16 (6)	67.33	±	3.38	5 (3)	1.82	±	0.34	5	4.19	±	0.53
Iris hermona	SMK	5 (3)	81.12	±	7.14	3 (2)	1.46	±	0.31	3	6.89	±	0.52
Iris lortetii	KDS	1 (1)	64.99	±	--	1 (1)	1.52	±	--	1	1.83	±	--
	MLK	1 (1)	68.48	±	--	1 (1)	1.54	±	--	1	3.46	±	--
	USA	1 (1)	88.45	±	--	1 (1)	1.27	±	--	1	3.69	±	--
Iris mariae	HAL	8 (5)	41.48	±	1.66	4 (1)	1.58	±	0.19	4	2.77	±	0.11
	I_maria	6 (2)	55.55	±	3.37	3 (2)	1.47	±	0.13	3	2.42	±	0.46
	NYZ	1 (1)	45.23	±	--	1 (1)	0.93	±	--	1	2.31	±	--
	SHV	15 (3)	46.03	±	1.08	5 (3)	1.72	±	0.27	5	3.21	±	0.31
	SKR	10 (5)	41.07	±	1.85	6 (2)	1.36	±	0.11	6	2.14	±	0.34
Iris petrana	DMN	12 (5)	38.95	±	1.79	5 (3)	1.19	±	0.13	5	4.59	±	0.35
	YER	12 (6)	35.92	±	1.53	4 (1)	0.80	±	0.04	4	3.34	±	0.74
	YMN	10 (6)	31.68	±	3.09	1 (1)	0.20	±	--	1	2.02	±	--
**Natural populations dataset**													
Iris atropurpurea	KUR	48 (15)	42.56	±	1.14	48	1.88	±	0.10	48	3.07	±	0.17
	NET	30 (13)	64.10	±	2.09	30	3.26	±	0.27	30	4.93	±	0.27
Iris petrana	YER	41 (23)	31.69	±	1.66	41	0.71	±	0.09	41	3.24	±	0.17
